# Ultrasound-guided stenting in the common femoral vein for accurate stent distal landing

**DOI:** 10.1016/j.jvscit.2023.101245

**Published:** 2023-06-17

**Authors:** Yuji Hoshino, Hiroyoshi Yokoi

**Affiliations:** a Section of Vascular Surgery, Fukuoka Sanno Hospital, Fukuoka, Japan; b Division of Cardiovascular Medicine, Fukuoka Sanno Hospital, Fukuoka, Japan

**Keywords:** Chronic venous insufficiency, Iliocaval venous obstruction, Post-thrombotic syndrome, Ultrasound-guided, Venous stent

## Abstract

**Objective/Background:**

Venous stenting has been reported with excellent clinical results; however, inadequate inflow can increase the risk of stent occlusion. When extending the stent into the common femoral vein (CFV), it is essential to ensure adequate inflow from the femoral vein, deep femoral vein (DFV), and great saphenous vein. Accurate identification of the distal landing zone (DLZ) of the stent is crucial to ensure adequate inflow. The DLZ is usually determined by venography or intravascular ultrasound (IVUS) with reference to bony landmarks. However, the uncertainty can lead to misidentification of the DLZ and inadequate stent placement, resulting in stent occlusion.

**Methods:**

From December 2016 to December 2022, 42 venous stent placements were performed in 40 patients with post-thrombotic iliofemoral vein obstruction and/or stenosis. Three patients had developed early stent occlusion owing to a misidentified DLZ. To improve accuracy in identifying the DLZ during venous stenting, ultrasound-guided placement was performed in the CFV of five patients (four on the left and one on the right) with post-thrombotic changes in the CFV and occlusion of the common iliac vein and external iliac vein. The distal end of the stent was adjusted just above the saphenofemoral junction in two cases and just proximal to the DFV confluence in three cases. Stent placement was verified using both long-axis ultrasound and fluoroscopy.

**Results:**

Ultrasound images of the CFV region provided clear visualization of the stent deployment site and accurate landmark locations, such as the saphenofemoral junction and DFV confluence, allowing for precise adjustments during stent deployment. This technique enabled easier and more definitive identification of other branches of the CFV than previously provided by IVUS and venography. No complications were observed in any of the 42 cases, and long-term patency was achieved at the final follow-up after stenting (average, 10 months; range, 3-14 months).

**Conclusions:**

Ultrasound-guided stenting in the CFV allows for real-time and accurate stent deployment with precise adjustment to the optimal DLZ. Using this technique, combined with venography and IVUS, missed distal lesions and subsequent stent occlusion can be prevented, potentially contributing to better treatment outcomes.

Venous stenting is increasingly used to manage femoral-iliocaval venous outflow obstruction or stenosis caused by post-thrombotic syndrome. Many studies have shown good technical results, excellent clinical outcomes, and a high patency rate; however, a higher risk of stent occlusion due to insufficient inflow has also been reported.^1^, ^2^, ^3^, ^4^, ^5^, ^6^, ^7^

Inflow into the common femoral vein (CFV) includes the femoral vein (FV), deep FV (DFV), and great saphenous vein (GSV). It is important to ensure sufficient blood flow from these veins as much as possible. In particular, blood flow from the DFV is crucial, and Williams and Dillavou^8^ have noted that care should be taken not to "jail" the DFV when stenting in the CFV.^6^ In addition to ensuring adequate inflow, stenting all areas of disease is crucial during venous stenting.^8^, ^9^, ^10^, ^11^ Identifying an accurate distal landing zone (DLZ) during a venous stenting procedure is essential to achieving these key elements. Typically, the DLZ is determined by venography and intravascular ultrasound (IVUS) using adjacent bony landmarks as a reference. However, misidentification of the DLZ due to inaccuracy can lead to inadequate stenting and is a major cause of stent occlusion.^1^^,^^12^^,^^13^ Precise identification of the femoral confluence remains a difficult, yet unsolved, technical challenge in venous stenting.^13^

To improve the accuracy of DLZ identification during venous stenting in the CFV, we performed ultrasound-guided stent placement. This technique allowed for easier identification of all branches of the CFV and more precise stent deployment with fine adjustments, potentially contributing to better outcomes in venous stenting.

## Methods

### Occurrence of acute stent occlusion due to missed DLZ and ultrasound-guided stenting

**Fig 1:**
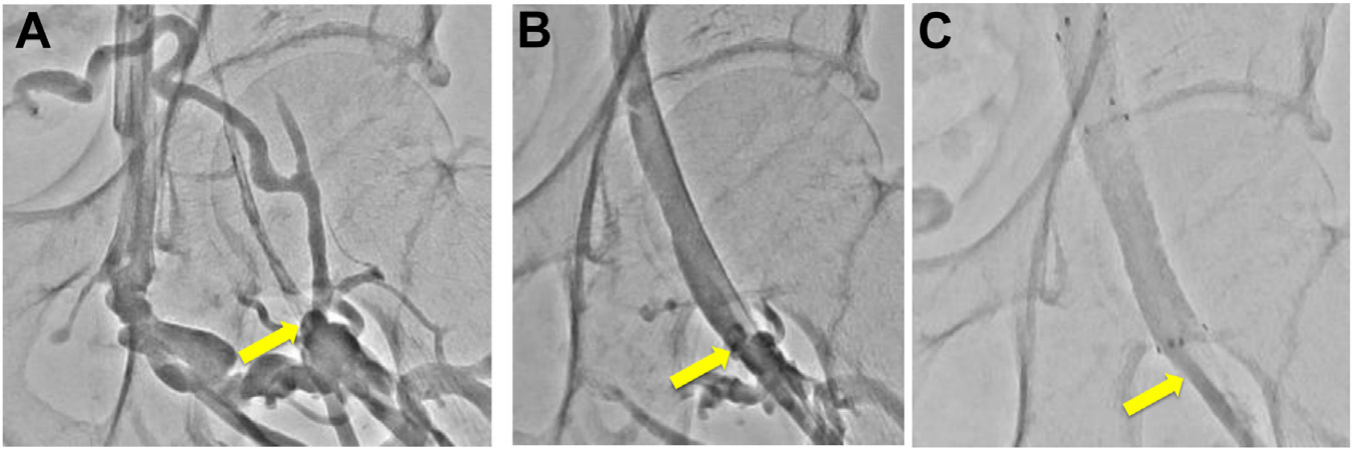
Venography findings of the original procedure in a patient with early stent occlusion on postoperative day 1. **A,** Initial venography findings demonstrating that the lesion in the left common femoral vein (CFV) is located below the bottom of the left femoral head (*arrow*). The developed collateral vessels obscure identification of the CFV in the frontal view. **B,** Venography findings after balloon predilation showing that the stenosis in the left CFV has been dilated (*arrow* indicates the same lesion site in the left CFV seen in **A**). **C,** Venography after stent placement showing the distal end of the stent located at the left femoral head. The lesion site observed below the bottom of the femoral head (*arrow*) was not fully covered by the stent (*arrow* indicates the same site in **A**).

Between December 2016 and December 2022, 42 iliofemoral venous stenting procedures were performed for 40 symptomatic patients with post-thrombotic iliofemoral vein obstruction and/or stenosis. Patient selection and the technical details of stenting have been previously described.^14^ Early stent occlusion was observed in three cases the day after stent implantation. Although these patients were asymptomatic, routine contrast-enhanced computed tomography and duplex ultrasound examinations revealed acute stent occlusion. In these cases, the native lesions involved occlusion of the common iliac vein (CIV) and external iliac vein (EIV) and post-thrombotic lesions extending to the CFV. The original procedures involved iliofemoral venous stenting, including the CFV region, with determination of the DLZs using venography and IVUS with the related bony landmarks. However, in all three patients, the DLZ was misidentified, resulting in incomplete coverage of the CFV lesion and early stent occlusion. Only balloon dilatation was performed in the distal part of the CFV, potentially leading to recoil and poor inflow of the lesion (Fig 1). Reinterventions, including pharmacomechanical thrombectomy and caudal stent extension, were performed in all cases, resulting in subsequent long-term patency. These experiences highlight the need for more precise identification of the DLZ during venous stenting procedures.

### Ultrasound-guided stent placement in the CFV

**Fig 2:**
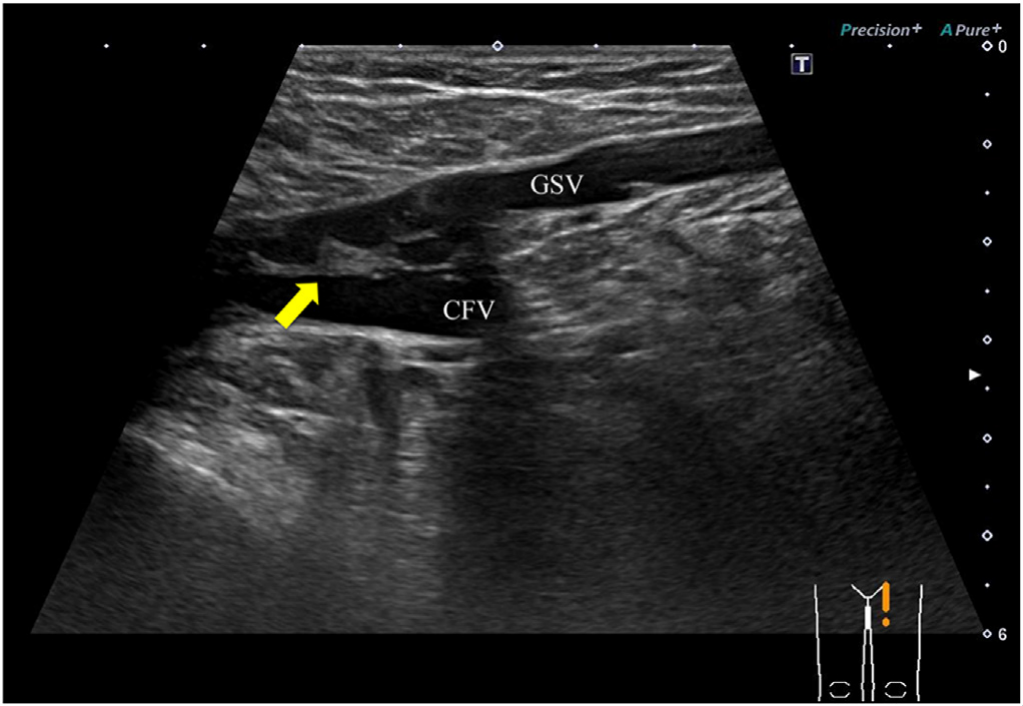
Ultrasound image showing post-thrombotic trabeculae (*arrow*) in the common femoral vein (CFV) extending to the saphenofemoral junction (SFJ). *GSV,* Great saphenous vein.

**Fig 3:**
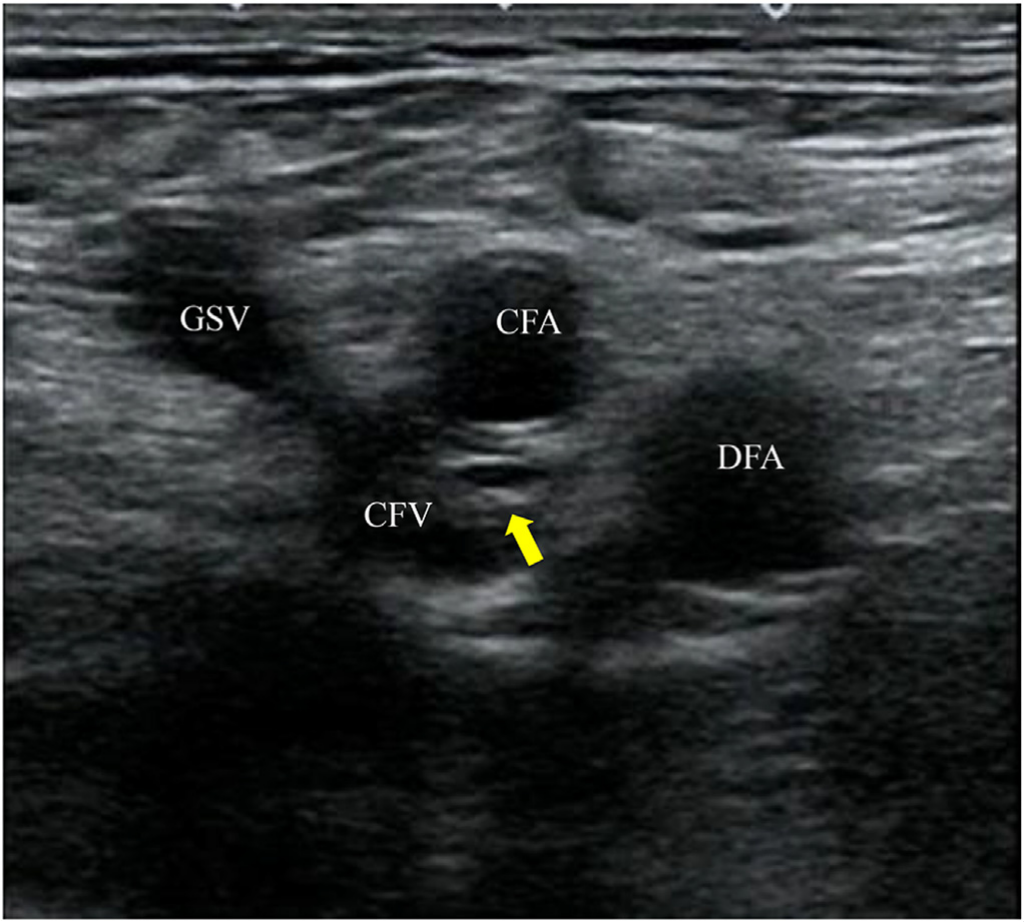
Ultrasound images showing post-thrombotic changes in the common femoral vein (CFV). Trabeculae in the CFV are observed below the saphenofemoral junction (SFJ) and above the deep femoral vein (DFV) bifurcation. *CFA,* Common femoral artery; *DFA,* deep femoral artery; *GSV,* great saphenous vein.

From January 2022 to December 2022, ultrasound-guided stenting was performed in the CFV of five patients (two men and three women; mean age, 59 years) for a more precise stent distal landing. All five patients had symptomatic post-thrombotic lesions, such as occlusion and/or stenosis and trabeculae, in the CFV, with CIV and EIV occlusion requiring iliofemoral venous stenting, including the CFV region. Of the lesions, four were in the left lower extremity and one was on the right side. One patient had C4b disease and four had C6 disease. Post-thrombotic changes in the CFV were classified into two types: those extending to the saphenofemoral junction (SFJ; three cases; Fig 2) and those beyond the SFJ and above the DFV bifurcation (two cases; Fig 3). Two patients had partial occlusion and stenosis in the FV, and none of the five patients had lesions in the DFV or GSV. The right internal jugular vein and an ipsilateral FV approach were used for four patients and the transjugular approach only was used for one patient. A 0.035-in. Glidewire (Terumo) was used to cross the lesions. The entire diseased areas from the CIV to the CFV were predilated with a 14-mm balloon (XXL Balloon Dilation Catheter; Boston Scientific Corp) and stented with a 14-mm S.M.A.R.T. Vascular Stent System (Cordis Corp) in the CIV and a 12-mm E-Luminexx (Bard Peripheral Vascular) in the EIV. The stents were then postdilated with a 14-mm balloon. The technical details of the procedure have been previously described.^14^ Following these procedures, stents were placed in the CFV under ultrasound guidance.

**Fig 4:**
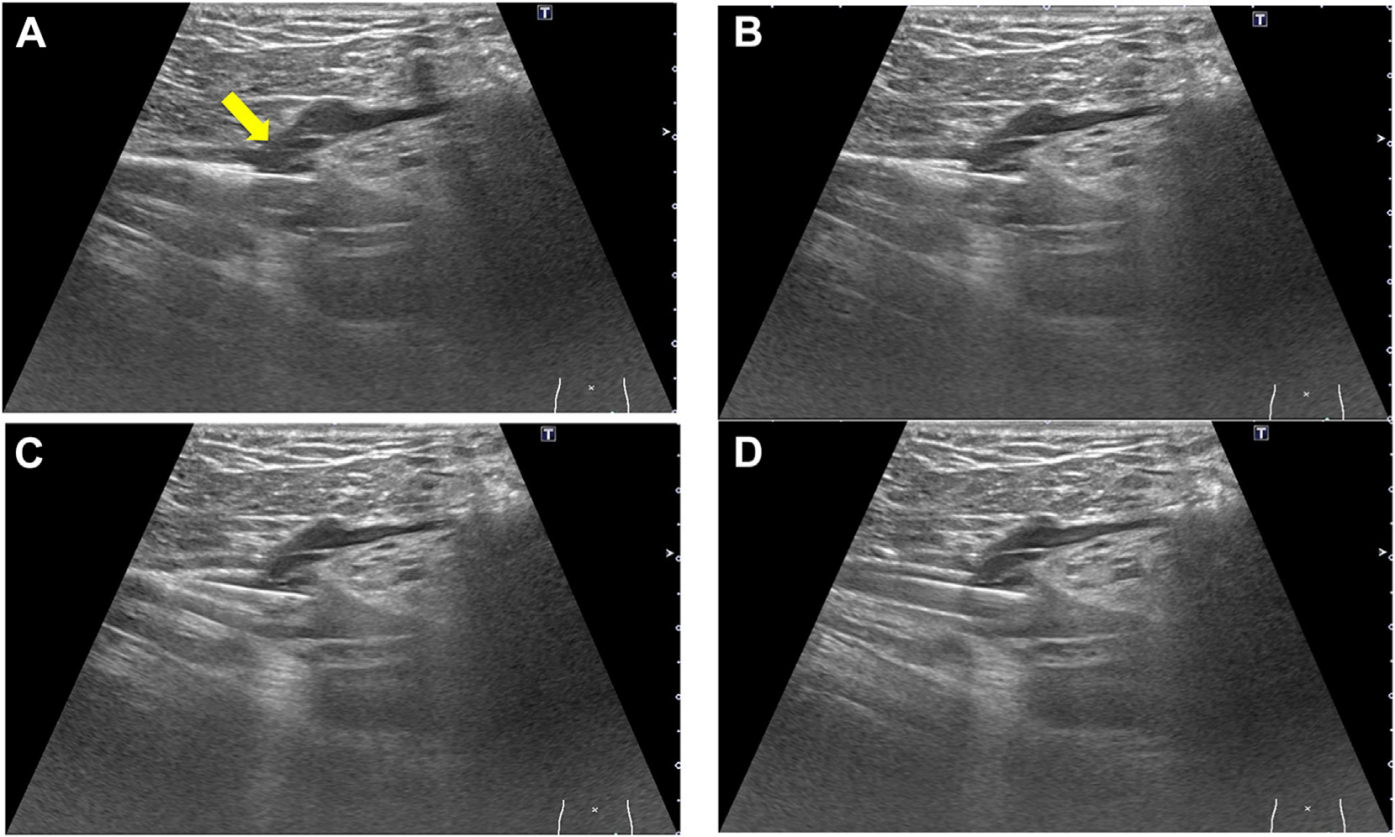
Ultrasound-guided stenting in the common femoral vein (CFV) just above the saphenofemoral junction (SFJ). **A,** The distal end of the stent was positioned just above the SFJ (*arrow*) under ultrasound guidance. **B-D,** The stent was deployed with careful adjustment of the distal end.

**Fig 5:**
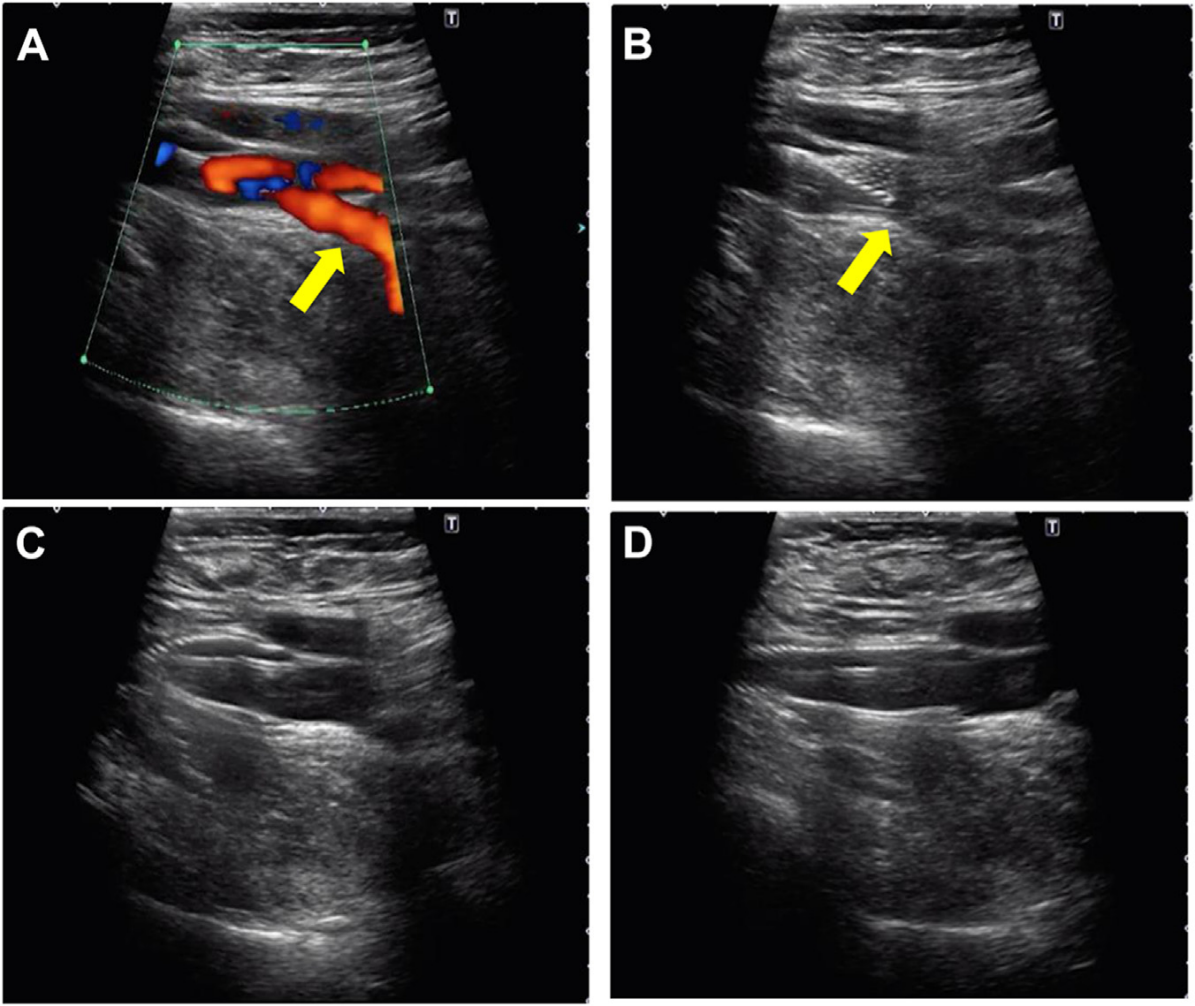
Ultrasound-guided stenting in the common femoral vein (CFV) just above the deep femoral vein (DFV) confluence. **A,** Color Doppler ultrasound showing blood flow from the DFV (*arrow*). **B,** The distal end of the stent was positioned just above the DFV confluence (*arrow*) under ultrasound guidance. **C,D,** The stent was deployed with careful adjustment of the distal end.

The guidewires from the transjugular approach were passed to the distal FV, and the CFV areas were visualized by ultrasound (Aplio-i700; Canon Medical Systems), which showed the location of the SFJ, confluence of the DFV, and other branches. The distal end of the stent was adjusted just above the SFJ in the three patients with post-thrombotic lesions extending to the SFJ (Fig 4; Supplementary Video 1, online only). For the two patients in whom the post-thrombotic changes extended beyond the SFJ into the CFV, the distal end of the stent was positioned just proximal to the confluence of the DFV (Fig 5; Supplementary Video 2, online only). Stenting of the CFV was performed with a 10-mm Wallstent (Boston Scientific Corp), and placement of the stent was verified using both long-axis ultrasound imaging and fluoroscopy. The DLZ was adjusted as necessary. In cases in which a gap remained between the EIV and CFV stents after postdilation of the CFV stent, an additional 12-mm stent was deployed to bridge the gap.

### Ethics

The medical ethics committee of Fukuoka Sanno Hospital approved the study (approval no. 20-FS-467), and all the patients provided written informed consent for all procedures and the report of their case details and imaging studies.

## Results

The use of ultrasound in stent deployment was similar to the technique commonly used for venous access. Ultrasound images of the CFV region provide clear visualization of the stent deployment site and accurate landmark locations, such as the SFJ and DFV confluence, allowing for precise adjustments during stent deployment. Furthermore, this technique enables easier and more definitive identification of other branches of the CFV, reducing the time, effort, contrast material use, and radiation exposure required to identify branch vessels, previously identified using IVUS and venography (data not measured). No complications were observed in any of the five patients, and all patients showed symptom improvement, including ulcer healing. Long-term patency was present at the final follow-up after stenting without any evidence of stent occlusion (mean, 10 months; range, 3-14 months). Because of the limited number of cases and short follow-up period, it was challenging to perform a statistical comparison of the patency rates before and after ultrasound-guided stenting. However, no acute stent occlusions due to missed inflow have occurred since implementing this approach.

## Discussion

In the present study, we have shown the advantages of ultrasound-guided stent placement, including precise deployment of the stent in the CFV and optimal DLZ adjustment. Inflow into the CFV includes the FV, DFV, and GSV, and ensuring adequate blood flow from these vessels is crucial in venous stenting.^3^^,^^6^ Therefore, the SFJ and DFV confluence are essential anatomic landmarks, and it is especially important not to jail the DFV orifice with the stent.^8^ Placement of the distal end of the stent is usually determined by venography and IVUS and adjacent bony landmarks. However, a study by Montminy et al^13^ revealed that the agreement between venography and IVUS in identifying the DLZ location was only 26%, highlighting the challenge of accurately identifying the femoral confluence during venous stenting. Several factors contribute to this difficulty, including the challenge of performing venography on the GSV and DFV, which are difficult to opacify. Even if they can be opacified, identification of their bifurcations is difficult. Additionally, developed collateral vessels can obscure the CFV trunk, and branches such as the lateral and medial femoral circumflex veins are also secondarily enlarged, further complicating the identification process. Furthermore, IVUS cannot evaluate the direction of the branching vessels, leading to uncertainty in identifying which branch corresponds to which vessel. Thus, identifying all branches of the CFV using venography and IVUS often requires significant time and effort because of the uncertainties involved. Misidentification of the proper DLZ can lead to inadequate stenting of existing venous lesions, potentially resulting in stent occlusion.^1^^,^^12^^,^^13^ Accurately deploying stents into the short segment between the SFJ and DFV confluence, which can be as short as 4 cm in some cases,^15^ is challenging when relying on adjacent bony landmarks.

Ultrasound imaging of the femoral vein region is commonly performed and provides a simple and definite evaluation of CFV lesions, the SFJ, the DFV confluence, and other branches. It is also frequently used for venous access during interventional procedures.

Ultrasound-guided stenting in the CFV allows for real-time and accurate stent deployment with precise adjustment to the optimal DLZ, effectively achieving both “ensuring adequate inflow” and “stenting all areas of disease,” which are key elements of successful venous stenting. Using this technique, in combination with venography and IVUS, missed distal lesions and subsequent stent occlusion can be prevented, potentially contributing to better treatment outcomes.

The technique requires a transjugular approach, which necessitates determining the area of stent placement and an appropriate DLZ in advance. A detailed preoperative assessment, including imaging studies such as ultrasound and computed tomography, can help identify the location and extent of venous lesions and the optimal DLZ for stent placement. This information can then be used to plan the procedure and achieve the best possible outcome. Although the final determination of the DLZ should be done using the intraoperative findings, it is essential to note that the vascular configuration can change with balloon predilation and vasospasm during the procedure, potentially resulting in missed lesions if overreliance is placed on the intraoperative imaging findings alone during venous stent placement.^12^^,^^16^

Missed lesions at the inflow site have been reported as a major cause of stent occlusion.^1^^,^^6^^,^^12^^,^^17^ In our study, the lesion was not fully covered by the stent, and only balloon dilation was performed, which could result in recoil and poor inflow. The effectiveness of caudal stent extension in achieving subsequent long-term patency supports this finding. Neglén et al^2^ reported that infrastent post-thrombotic disease can result in poor venous outflow of the limb, decreasing flow into the stent and contributing to thrombosis of the stent. Therefore, precise preoperative assessment of the lesion is essential to avoid such complications. Recent advances in diagnostic imaging technologies have allowed for a more detailed delineation of the disease, aiding in accurately determining the optimal treatment strategy.

## Conclusions

Ultrasound-guided stenting in the CFV allows for real-time and accurate stent deployment with precise adjustment of the optimal DLZ, potentially contributing to better treatment outcomes. Precise preoperative assessment of the lesion in finer detail can provide a more comprehensive evaluation of the disease, helping to plan the procedure for the best possible outcome. Using these techniques, in combination with venography and IVUS, missed distal lesions and subsequent stent occlusion can be prevented, ultimately improving the success rate of venous stenting.

## Appendix (online only)

Supplementary Video 1 (online only) Ultrasound-guided stenting in the common femoral vein (CFV) just above the saphenofemoral junction (SFJ).

Supplementary Video 2 (online only) Ultrasound-guided stenting in the common femoral vein (CFV) just above the deep femoral vein (DFV) confluence.
